# Behavioural effects of the common brain-infecting parasite *Pseudoloma neurophilia* in laboratory zebrafish (*Danio rerio*)

**DOI:** 10.1038/s41598-020-64948-8

**Published:** 2020-05-15

**Authors:** Helene L. E. Midttun, Marco A. Vindas, Lauren E. Nadler, Øyvind Øverli, Ida B. Johansen

**Affiliations:** Norwegian University of Life Sciences, Faculty of Veterinary Medicine, Department of Paraclinical Sciences, P.O. Box 369, Sentrum N-0102 Oslo, Norway

**Keywords:** Infectious diseases, Animal behaviour, Diseases of the nervous system, Infection

## Abstract

Research conducted on model organisms may be biased due to undetected pathogen infections. Recently, screening studies discovered high prevalence of the microsporidium *Pseudoloma neurophilia* in zebrafish (*Danio rerio*) facilities. This spore-forming unicellular parasite aggregates in brain regions associated with motor function and anxiety, and despite its high occurrence little is known about how sub-clinical infection affects behaviour. Here, we assessed how *P. neurophilia* infection alters the zebrafish´s response to four commonly used neurobehavioral tests, namely: mirror biting, open field, light/dark preference and social preference, used to quantify aggression, exploration, anxiety, and sociability. Although sociability and aggression remained unaltered, infected hosts exhibited reduced activity, elevated rates of freezing behaviour, and sex-specific effects on exploration. These results indicate that caution is warranted in the interpretation of zebrafish behaviour, particularly since in most cases infection status is unknown. This highlights the importance of comprehensive monitoring procedures to detect sub-clinical infections in laboratory animals.

## Introduction

Model animal species (e.g., rodents, invertebrates and fish) are widely used in biomedicine, where study outcomes hinge on reproducibility of the results. Regular health monitoring of these animals has improved over time, as parasites and pathogens (*e.g.* microparasites, macroparasites, bacteria, viruses) are known to influence animal physiology, immune mechanisms, functional morphology, behaviour, and welfare^1,2^. However, monitoring procedures may fail to detect subclinical infections (*i.e*., exhibiting no external signs of disease), in animals that appear otherwise healthy^3^. Thus, undetected infections can inadvertently bias results obtained from these studies, which has repercussions on many research areas, such as biomedicine. The scale of this issue is only just being uncovered. In rodents, for example, Pritchett-Corning *et al*.^4^ reported the prevalence of sixteen commonly undetected pathogens in mice and rats from pharmaceutical, biotechnology, academic, and governmental institutions in North America and Europe. However, the practical impacts of these elusive infectious agents on frequently used experimental assays remain largely unknown.

Undetected parasites and pathogens can alter experimental results in model organisms in several ways. Many species of parasites seem to be particularly adapted to affect host neuroendocrine signalling and behaviour in ways which enhance parasite fitness^5–7^, but other aspects of host phenotype are indeed also affected by infection. For example, the intracellular parasite *Wolbachia*, which is commonly found in laboratory *Drosophila* spp. colonies, can reduce host egg viability, confound host optimal trait expression (*i.e*., intra-locus sexual conflict) and alter host circadian rhythms^8–10^, all commonly measured traits in biomedical studies. Similar effects have been observed in rodent model systems. A common infectious agent in rodent facilities is the pathogen murine norovirus^4,11^, which can induce tissue inflammation and activate cytokine signalling in murine macrophages^12–14^. In well-established model animal systems, like *Drosophila* spp. and rodents (*e.g*. *Mus musculus*, *Rattus norvegicus*), substantial efforts in recent years have focused on how common parasites and pathogens spread within and among laboratory facilities, as well as best practices to remove these infectious agents once established. This work has helped to successfully eliminate and prevent many infections from research facilities, improving both animal welfare and the reproducibility of study outcomes^15,16^. However, in newer model organism species (*e.g*., zebrafish, *Danio rerio;* medaka, *Oryzias latipes*; goldfish, *Carassius auratus*), data on the prevalence of pathogens in laboratory colonies and their potential confounding effects remain limited.

The use of zebrafish as a model organism has boomed in recent years, first gaining momentum in the 1990’s^17^. Due to the relatively short time period since zebrafish were introduced as a model organism, there is a scarcity of research on the pathogenesis of common infectious agents in this species. Furthermore, standard health monitoring programmes to prevent the introduction of pathogens in zebrafish facilities are not widely practiced^18–20^. In fact, many zebrafish facilities do not screen for pathogens. Further, sometimes zebrafish bought at commercial pet stores are introduced into zebrafish facilities without prior comprehensive pathogen screening^21^. One of the most common diagnoses in zebrafish submitted for health monitoring to the Zebrafish International Research Center (ZIRC) is infection with the microsporidian parasite *Pseudoloma neurophilia*. Depending on the year, more than 50% of these facilities test positive for *P. neurophilia* annually^22^. This parasite takes advantage of both horizontal (*i.e*., transmission between conspecifics following contact) and vertical (*i.e*., transmission from mother to offspring) transmission. Infection spreads mainly through ingestion of the infectious spore stage. Spores are released to the water from dead infected hosts or with feces and during spawning^23,24^. Infections are largely subclinical, and are often only detected in severe cases, when hosts develop spinal deformations and emaciation^25,26^. *Pseudoloma neurophilia* primarily infects the hindbrain and the spinal nerve roots of the spinal cord^27^, areas commonly associated with motor function, freezing, fear-learning and anxiety^28,29^. Whether *P. neurophilia* alters emotional states like fear and anxiety in zebrafish has been suggested but remains little explored. If so, laboratories using zebrafish as animal model to study these emotional states could be critically affected by the presence of the parasite.

Recent studies report that *P. neurophilia*-infection alter startle responses (*i.e*. response to fearful stimuli)^30^ and increase shoal cohesion (*i.e*. reduced inter-fish distances) in zebrafish^31^. These behavioural changes were interpreted as a parasite-induced increase in stress, fear and anxiety. It can, however, be challenging to extrapolate emotional states like fear and anxiety from behavioural outputs such as shoal cohesion. For example, the increase in shoal cohesion was interpreted as a stress/anxiety response to infection^31^, but could might as well reflect increased sociability^32^ or even a reduction in locomotion^33^. Thus, in order to understand how this parasite affects major behavioural outputs (*e.g*. anxiety, sociability, aggression) commonly studied by the zebrafish community, individual behavioural effects of parasite infection should be investigated across a range of contexts and preferably by using the most common neurobehavioral assays.

**Figure 1:**
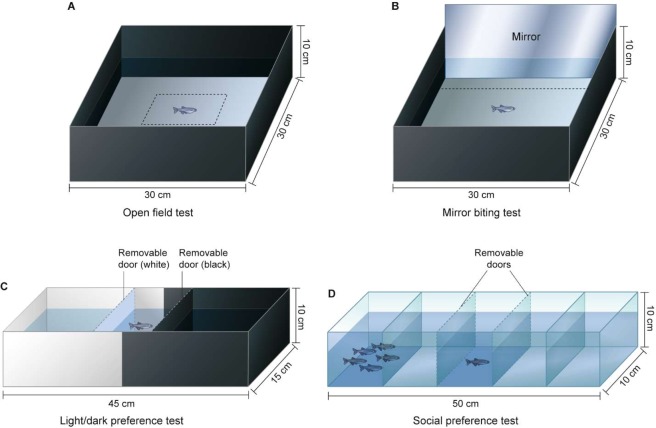
Behavioural test arenas. (**A**) In the open field test, zebrafish were transferred to the arena and allowed to explore for five minutes. The following behavioural outputs were measured: freezing, thigmotaxis and time spent in the centre of the arena. (**B**) In the mirror biting test, zebrafish interact with their mirror image, with aggression quantified as the amount of times the zebrafish attacks its own mirror image within six minutes. (**C**) For the light/dark preference test, zebrafish acclimated between removable doors for five minutes, before doors were removed. The fish was then able to move freely in the arena for 15 minutes. Crossings between compartments and scototaxis were measured. (**D**) In the social preference test, five conspecifics were placed in one chamber of the arena, while the chamber at the opposite end remained empty. The zebrafish acclimated for five minutes between the removable doors, before it was allowed to freely explore the arena for a total of 11 minutes. Time spent in all compartments and total distance moved were measured.

Here, we employed four commonly used tests to examine how *P. neurophilia* infection in zebrafish influences the following behavioural outputs: aggression, sociability and anxiety (*i.e*., open field, mirror biting, light/dark preference, social preference). For all tests, we compared individual locomotor function and general activity in infected and uninfected fish. We hypothesised that *P. neurophilia* infection affects behavioural outputs associated with anxiety and/or sociability, given the location of the parasite in the hindbrain, and based on previous findings^27,30,31^. In the open field test (Fig. 1A), thigmotaxis (*i.e*., maintaining proximity to the wall of an experimental arena) is quantified as a proxy for anxiety, while exploration (*i.e*., moving in the centre of the arena) is interpreted as boldness^34,35^. In the mirror biting test (Fig. 1B), biting at or interacting with the mirror image is interpreted as aggression^36,37^. In the light-dark preference test (Fig. 1C), scototaxis (*i.e*., aversion of bright places) is commonly interpreted as anxiety (Araujo *et al*.^38^). Lastly, the social preference test (Fig. 1D) assesses sociability, by examining an individual’s tendency to associate with conspecifics versus remaining solitary^37^. In sum, there is potentially high overlap between the behavioural patterns and control systems typically addressed in biological studies, and those a parasite, pathogen, or components of the microbiome might adaptively target. Hence, by studying behavioural variation between infected and uninfected zebrafish, we can gain a better insight into how research outcomes vary with infection status, and the importance of identifying and characterizing pathogens vs commensals in model organisms.

## Results

**Figure 2:**
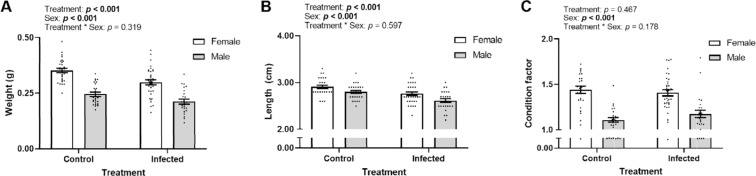
Weight (**A**), length (**B**) and Fulton’s K condition factor (CF, **C**) of zebrafish (*Danio rerio*) experimentally infected with the microsporidian parasite *Pseudoloma neurophilia* (“Infected”) or sham infected (“control”) for 10 weeks. Weight and length were obtained post mortem, from which CF was calculated. All bars indicate the mean ± SEM (generalized linear model analysis). Dots indicate individual data points (n_control_ = 57, n_infected_ = 60). All graphs obtained using GraphPad Prism v8.3.1^69^.

To verify infection status, we tested brain tissue from infected and uninfected fish for the presence of *P. neurophilia* by qPCR^39^. All tested fish in the parasite-exposed group tested positive for the parasite, including the random selection of fish tested already after 6 weeks of infection. Conversely, all tested fish from the uninfected treatment group were negative for infection (data pooled in Fig. S1). Fish harbouring *P. neurophilia* infection exhibited an approximately 13% lower body mass (Generalized linear model (GLM): F_1,115_ = 14.41, p = 0.0002, Fig. 2A) and 5% shorter body length (GLM: F_1,115_ = 20.55, p = 0.0002, Fig. 2B) than uninfected controls. However, Fulton’s K condition factor (a weight-length relationship that is used as a health status indicator in fish^40^) was not altered by infection status (p > 0.05, Table S1, Fig. 2C). Although all measures of size and condition differed significantly between males and females, the interaction between sex and infection was not significant for any of these variables (Table S1).

### *Pseudoloma neurophilia* infection has distinct effects on zebrafish behaviour

Four neurobehavioral assays were conducted in which the behaviour of infected versus uninfected fish were compared (the experimental arenas used for each test are illustrated in Fig. 1). Complete details of all statistical outputs and sample sizes are summarized in Table S1 and S2.

**Figure 3:**
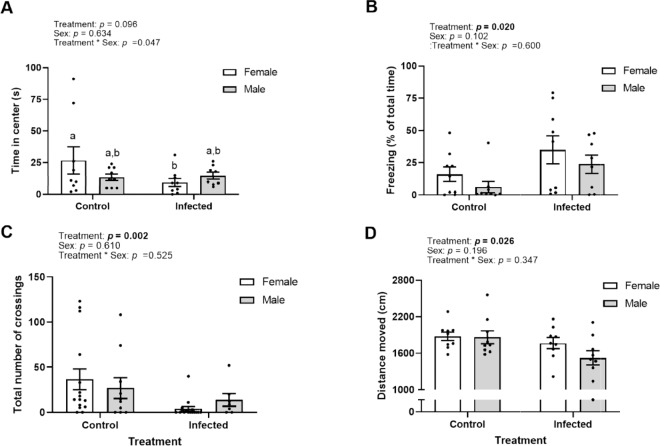
Behavioural effects of *Pseudoloma neurophilia*-infection in female and male experimentally infected zebrafish and uninfected controls. (**A**) Total time spent in the centre of the arena in the open field test (s ± SEM), p = 0.048, n_control_ = 18, n_infected_ = 17. (**B**) Average total time spent freezing as percent of all time in both the open field test and mirror biting test combined,* i.e*. 11 min (% ± SEM), p = 0.02, n_control_ = 18, n_infected_ = 17. (**C**) Total number of crossings between black and white compartment in the light/dark preference test (frequency ± SEM), p = 0.0018, n = 25 per treatment group. (**D**) Total distance moved in the social preference test (cm ± SEM), p = 0.026, n = 18 per treatment group. All graphs obtained using GraphPad Prism v8.3.1^69^.

*Pseudoloma neurophilia* altered time spent in the centre of the arena, *i.e*. exploration, in a sex-specific manner (Negative Binomial Generalized Linear Model, Infection*Sex Interaction: χ^2^_1_ = 3.92, p = 0.047). Although uninfected and infected males exhibited similar responses, uninfected females spent substantially more time on average in the centre of the arena, *i.e.* displaying exploration, than infected females (Tukey post-hoc test: p = 0.044, Fig. 3A).

Infection did not alter the number of bites towards the mirror image in the mirror biting test, *i.e*., aggression (Fig. S2a, Table S2). For both open field and mirror biting tests, *P. neurophilia* infected fish exhibited approximately three times more freezing behaviour, *i.e*., moving less than one body length/second (Negative Binomial Generalized Linear Mixed-Effects Model, Infection: χ^2^_1_ = 5.40, p = 0.020, Fig. 3B, Table S2), a trait typically associated with anxiety^41–43^. Neither aggression nor freezing behaviour differed significantly between the sexes (Table S2).

In the light-dark preference test, infection did not affect time spent in the white or dark compartment (Fig. S2b, Table S2). Both treatment groups spent on average approximately 50% of the trial period in each compartment. However, *P. neurophilia* infection reduced the number of crossings between compartments by five times (Zero-inflated Count Data Regression Model, Infection: χ^2^_1_ = 9.73, p = 0.002, Fig. 3C), a trend typically associated with an overall decrease in locomotor activity^44^.

Sociability, *i.e*., the preference to remain close to conspecifics, was not affected by *P. neurophilia* in the social preference test (Fig. S2c, Table S2), with zebrafish spending 86% of their time in proximity to conspecifics on average. However, *P. neurophilia* infection affected locomotor activity, which was evident by a 13% reduction in distance moved relative to uninfected controls (Generalized Linear Model, Infection: F_1,34_ = 5.49, p = 0.026, Fig. 3D). There was no effect of sex on either sociability or distance moved in this test (Table S1 and S2).

## Discussion

Our study demonstrates direct impacts of *P. neurophilia* infection on zebrafish behavioural responses to four commonly used tests in neurobehavioural studies. Therefore, this parasite could impact the reproducibility of study outcomes in a range of scientific fields, particularly because zebrafish infected with *P. neurophilia* exhibited reduced locomotor activity across a range of contexts. However, several behavioural traits were unchanged by infection, including sociability, aggression and thigmotaxis, indicating that in animals with unknown infection status, robust experimental results can still be gleaned with careful planning and analysis. These results illustrate the complex role of parasite infection in host behaviour and highlight the importance of examining behavioural phenotypes across several contexts to comprehensively characterize the impacts of parasite infection.

Infected individuals conducted fewer crossings between the white and black compartments in the light-dark preference test and moved a shorter distance in the social preference test, both commonly used indicators of locomotor activity^34,44,45^. These results are in line with previous literature on several host-parasite systems, in which parasite infection was associated with reduced locomotor activity^46–48^. Parasites can affect host locomotion through alterations in host morphology and/or physiology^49^. For example the trematode *Ascocotyle pachycystis* infects the heart and reduces swimming performances in the sheepshead minnow (*Cyprinodon variegatus*)^50^. In our study, we observed a reduction in body size (both length and mass) with infection, a trait that potentially could influence locomotion. However, Tran and Gerlai^45^ found that individual differences in locomotor activity do not shift substantially with length or weight in zebrafish, and thus is unlikely to be a major driving factor in our observed effect on locomotion. Instead, *P. neurophilia* may directly affect locomotion by infecting nerve tracts controlling motor function^27,30^, a scenario that deserves further investigation. Alternatively, a change in locomotor activity can be caused by the parasite’s effect on host energy metabolism and immune or endocrine function. Indeed, reduced locomotion and growth in infected zebrafish can represent subtle symptoms of sickness behaviour (characterized by lethargy, anxiety and anorexia), an adaptive and organized behavioural strategy aimed at for example conserving energy^51^.

Sickness behaviour is sometimes also characterized by anxiety^52^. Indeed, increased freezing behaviour observed with *P. neurophilia* infection in both the open field and the mirror biting tests (Fig. 3B) could reflect anxiety-like behaviour. Spagnoli *et al*.^27,30^ recently reported that *P. neurophilia* infects brain areas associated with anxiety and fear-learning^53^. However, freezing behaviour may not be a reliable measure of fear and anxiety in zebrafish since neither alarm pheromones nor the presence of predator cues increased freezing behaviour in zebrafish^43,54,55^. Alternatively, increased freezing in *P. neurophilia* infected zebrafish in our study could reflect immobility. In fact, the terms freezing behaviour and immobility are used interchangeably in the zebrafish literature and are difficult to differentiate^36^. Immobility is a well-known response to animal infection^56^ and in line with our observations of a general reduction in locomotor activity (*i.e*., crossings and distance moved, as described above).

If the increased freezing behaviour observed in the current study reflects a parasite-induced increase in anxiety-like behavior, we would expect reduced exploration (*i.e*. boldness) in the open field test. Although we found that *P. neurophilia* decreased exploration in female zebrafish hosts, exploration was not decreased in infected males. Exploration is a measure of boldness and risk-taking^57^, here measured as the time spent in the center of the open field arena. Our results could thus indicate that infection induces a sex-specific decrease in boldness. As exploration and boldness are commonly used traits in research, including *e.g*. biomedical studies, undetected infections could have broad implications for study outcomes, particularly in studies that use either just one sex or an unequal number of males and females across treatments. These results are also in agreement with previous literature suggesting that parasite infection affects male and female zebrafish differently. For example, recent studies show that male zebrafish are more susceptible to *P. neurophilia* infection and suffer from greater parasite clusters than females^27,58^. Conversely, infected females are thinner than uninfected individuals, which is associated with a reduction in ovary size and egg development. In fact, *P. neurophilia* infection has been shown to reduce condition factor (length:width ratio) in female, but not male, zebrafish^59^. In the current study we calculated condition factor based on weight instead of width and did not observe sex-specific effects of *P. neurophilia* on condition. Nevertheless, potential sex-differentiated effects of infection could ultimately introduce uncontrolled variation into study outcomes, particularly when only one sex is employed, further reinforcing the need to carefully plan study design when infection status is unknown.

We found no change in sociability in response to infection. Microsporidia-infected zebrafish and sticklebacks (*Gasterosteus aculeatus*) have previously been shown to swim in more cohesive shoals than uninfected groups^31,60^. For example, Spagnoli *et al*.^31^ found that *P. neurophilia* increases shoaling cohesion in zebrafish. Increased shoaling could reflect either stress/anxiety, increased sociability or reduced locomotion^31,32^. By studying sociability in the social preference test, we found that uninfected and infected zebrafish were uniformly social, spending 86% of their time on average with conspecifics. It is however possible that the increased shoal cohesion observed previously^31^ could be the result of stress/anxiety or the general reduction in activity levels in infected zebrafish that we observe. In the Qingbo carp (*Spinibarbus sinensis*), shoal cohesion increases with decreasing locomotor activity, likely because individual repulsion radiuses increase at higher swimming speeds^33^. As illustrated by the above example, the fact that *P. neurophilia* appears to have a general effect on activity may obscure conclusions regarding the effect of this parasite on motivational or emotional states like sociability. Regardless of what mechanism increases shoal cohesion, it is tempting to speculate that a greater shoal cohesion could increase transmission rates for the parasite. Indeed, transmission of parasites with direct life cycles has previously been shown to increase with closer shoal formations in other taxa^61^.

Taken together, our results illustrate that subclinical, and therefore often undetected infections, result in the alteration of behavioural outputs in a context and sex-specific manner. We show that infection alter locomotion but may also induce anxiety-like behaviour. Moreover, the parasite may also affect the behaviour of male and female hosts differently, with important implications for the reproducibility of results in studies using this model system. Since *P. neurophilia* infection appears to influence primarily energetically costly processes including growth (Fig. 2), locomotion (Fig. 3), fecundity^62^ and gonad development^59^, future studies should focus on effects of *P. neurophilia* infection on host energetics and characterise any costly biological processes which are stimulated by infection (*e.g*., immune responses). Laboratory animals are still crucial for the scientific world, thus parasitic infections in zebrafish presents concerns for both animal welfare as well as reproducibility and hence impact of neurobehavioural studies.

## Materials and Methods

### Ethics

This work was approved by the Norwegian Animal Research Authority (NARA), following the Norwegian laws and regulations controlling experiments and procedures on live animals in Norway (permit number 11241).

### Fish husbandry

All experiments were performed at the Norwegian University of Life Sciences, campus Adamstuen (Oslo, Norway). Ten adult AB zebrafish (5 males and 5 females) were obtained from the Sinnhuber Aquatic Research Laboratory (SARL) at Oregon State University, a *P. neurophilia* specific pathogen free (SPF) facility. Fish were kept in a quarantine room in a 25 L tank (40 cm × 25 cm × 25 cm; L × W × H) for an acclimation period of two months. The tank was provided with filtered and UV-treated water. In addition, 50% of the water in the tank was changed twice weekly in order to further maintain high standards of water quality. Water temperature was maintained at 28 °C, pH and conductivity were kept at 7.4–7.6 and 500 µS respectively, following husbandry practices recommendations by ZIRC^63^. All fish were fed flake food twice daily (Special Diets Service, Witham, Great Britain) and live brine shrimp (Ocean Nutrition, Essen, Belgium) once per day. After two months, all fish were transferred to a standardized recirculation system, where they were kept at a density of 5 fish/L (Tecniplast, Buguggiate, Italy).

Once weekly, adult fish were placed overnight in standard 1 L crossing tanks for spawning (Techniplast, Buguggiate, Italy), with males placed at one side and females on the other side (with a 1:1 male:female ratio). The following morning, the divider was removed for up to four hours, allowing fish to spawn, according to ZIRC recommendations^63^. Following spawning, fish were placed back in their respective holding tanks and eggs were collected. Eggs were rinsed with filtered and UV-treated water, counted and maintained in petri dishes (95 ×15 mm; Heger, Rjukan, Norway) at a density of 50 eggs/30 mL at 28 °C until 5 days post fertilization (dpf). During this period, water was changed, and dead eggs were removed daily. At five dpf, zebrafish larvae were transferred to 1 L plastic beakers (VWR, Radnor Pennsylvania, USA), at a density of 1 fish per 6 mL of filtered and UV-treated water. Two times per day larvae were fed freeze dried rotifers and small-grained dry food (Special Diets Service, Witham, United Kingdom). Water was changed daily. At 21 dpf, juvenile zebrafish were transferred to a recirculating aquarium system in which conditions (*i.e*. pH, salinity, temperature and water quality) and feeding routine were kept as described above. The light:dark cycle was always kept at 14 h light:10 h dark.

### Infection protocol

At approximately 5 months post-hatch, 252 zebrafish were transferred from the F1 generation to an infection room. Here, the zebrafish were housed in 30 closed-tanks (23 ×15.3 ×16.5 cm, L x W x H) (Exo Terra, Montreal, Canada) at a density of 5 fish/L. We randomly assigned the zebrafish to treatment groups and tanks using a random number generator (https://www.random.org/), keeping a female:male ratio of 1:1 in each group. Water temperature was maintained at 26–28 °C and the water was aerated continuously, with 50% water changes conducted three times weekly and 100% water changes once biweekly. Concurrently, *P. neurophilia*-infected zebrafish from the Norwegian University of Life Sciences (NMBU) zebrafish facility were maintained in a 25 L tank (40 cm ×25 cm ×25 cm; L x W x H). The NMBU facilities did not test positive for *Mycobacterium* spp. during routine screenings of water samples and has no known history of other pathogens. Positive infection for *P. neurophilia* in zebrafish at the facility was tested via qPCR as described below.

In order to reach an infection prevalence of approximately 100% in the infected treatment, experimental infections were conducted over a 10-week period. During this time, 100 mL of the home tank water was replaced with 100 mL of water from the tank containing *P. neurophilia*-infected zebrafish on a daily basis. In addition, zebrafish were fed central nervous system (CNS) tissue from infected conspecifics four times during the course of the infection study (with a minimum of two weeks between feedings), according to the infection protocol outlined in Peneyra *et al*.^64^. Briefly, macerated CNS from infected fish was mixed with zebrafish food and subsequently fed to the study fish. In the same manner, control fish received water from a tank with spf fish and CNS tissue from uninfected fish. During the infection period, a total of 14 fish died (12 from infected and 2 from control groups). Six weeks into the infection protocol we tested for the presence of *P. neurophilia* by randomly selecting one fish from each tank (n = 15 per group) by euthanizing the fish in an overdose of Tricaine methanesulfonate (1 g/L; MS-222; Sigma, St. Louis Missouri, USA), before dissecting out the whole brain. The brains were excised within 3 min and rapidly frozen on dry ice, then stored at −80 °C until further qPCR analysis for the presence of *P. neurophilia*. In addition, brain tissue from approximately 80% of experimental fish (tested in behavioural trials) was similarly stored and analysed for the presence of *P. neurophilia* after behavioural testing. Fish were not screened for the presence of other pathogens, as the SPF fish obtained from SARL were maintained in quarantine from the NMBU facility´s other zebrafish after arrival (in a separate room) in a new tank and then in a recirculating system that had not been used for fish husbandry prior to this study.

### DNA extraction and qPCR

Brain tissue from infected and uninfected fish was transferred to 50 µL MilliQ water (Merck, Darmstadt, Germany). Samples were sonicated for 2 minutes at 55 W (QSonica Sonicators, Connecticut Newtown, USA) and immediately placed on ice. The sonicator probe was decontaminated with 100% ethanol and MilliQ water between samples. The DNeasy Blood and Tissue Kit (Qiagen, Hilden, Germany) was used to extract DNA according to manufacturer’s protocol, with the addition of an overnight proteinase K and lysis buffer digestion at 56 °C, following the protocol outlined in Sanders and Kent^39^. Samples were then eluted in 100 µL storage buffer (provided in the kit). The qPCR protocol for analysis of infection status was established by Sanders and Kent^39^. Briefly, all reactions were performed in 25 µL, with forward and reverse primer concentrations of 900 nm each, 250 nM hydrolysis probe, 1X TaqMan and 2 µL DNA sample. Forward primer, reverse primers and hydrolysis probe used were 5′-GTAATCGCGGGCTCACTAAG-3′, 5′-GCTCGCTCAGCCAAATAAAC-3′ and 5′-6-carboxyfluorescein (FAM)-ACACACCGCCCGTCGTTATCGAA-3′-Black Hole Quencher 1 (BHQ1) respectively. The qPCR was performed using the following program: 50 °C for 2 minutes, 95 °C for 10 minutes followed by 40 cycles of 95 °C for 15 s, 60 °C for 1 minute on a LightCycler 96 instrument and analysed using the LightCycler 96 software (Roche, Basel, Switzerland).

Primers are species-specific for *P. neurophilia*, thus all expression indicates presence of the parasite, however Cq-values above 38 were too considered negative.

### Behavioural testing

Following 10 weeks of experimental infections, zebrafish were tested in one of the three test arenas described below. Each fish was only tested once. Only fish with subclinical infections (*i.e*. fish without scoliosis or any signs of emaciation) were used for behavioural testing. For all tests, behavioural experiments were video recorded from above, with arenas shielded from surroundings by black plastic while trials were conducted. All behavioural trials used filtered and UV-sterilised water maintained at 28 °C. Water was changed between each trial. All trials were performed between 09:00 and 14:00. All fish were euthanised immediately after each test as described above. Fish were measured for weight and length, which was used to calculate Fulton’s K condition factor (100*(weight/length3)).

#### Open field and mirror biting test

To assess anxiety, exploration and aggression, we used a combination of protocols for the open field test^35^ and for the mirror biting test^37^. It is generally assumed that, similar to rodents, zebrafish show a natural aversion for brightly lit open spaces, but simultaneously have a natural drive for exploring novel environments^65^. Thus, in the open field test, freezing behaviour (moving < 0.1 cm/s) and avoidance of the centre of arena is interpreted as anxiety-like behaviour. Conversely, visits to and time spent in centre of arena is classically interpreted as boldness and willingness to explore. In the mirror biting test, aggression is analysed by quantifying time tracing the mirror, frequency of mirror bites (i.e. biting or butting head at own mirror image) and latency to first attack (i.e., time it takes for the fish to conduct its first bite towards mirror). We tested 18 control and 18 *P. neurophilia*-infected fish. The test was performed in an apparatus measuring 30 × 30 × 10 cm (W × L × D), with black walls and a white bottom (Fig. 1A,B). The apparatus was filled with 4 L of water. Fish were video-recorded for 5 minutes after being placed in the area. Following the initial 5 min, a mirror was placed at one side of the arena, and fish were left to interact with their mirror image for a total of 6 minutes, while continuing to be video-recorded.

#### Light/dark preference

In zebrafish, scototaxis (*i.e*., the avoidance of bright places) is a behavioural correlate for anxiety^66^, with increased time in dark being associated with increased anxiety. To test whether *P. neurophilia* affects this trait in zebrafish, we performed a light/dark preference test, following the protocol by Araujo *et al*.^38^. A total of 25 *P. neurophilia*-infected and 25 control fish were tested. The apparatus (15 ×45 ×10 cm, W x L x D) was divided vertically into a black and a white half with removable doors in corresponding colours to their location (Fig. 1C). The apparatus was filled with 4 L of water. Fish were individually moved to the central compartment (*i.e*., between the removable doors) for a 5-min acclimation period, after which the doors were removed. Fish were then video-recorded for 15 mins. Water was changed between each trial. Time spent in white, freezing behaviour in white and total number of crossings between the dark and light compartments were recorded and analysed.

#### Social preference

Zebrafish actively form shoals, a trait that is attributed to social behaviour^67^. Thus, to test whether *P. neurophilia* affects sociability in its host, we performed a social preference test using the protocol developed by Pham *et al*.^37^. The protocol was followed with minor changes; briefly, a Plexiglas arena (10 × 50 × 15 cm, W × L × H) was divided into five compartments with transparent dividers. The three middle compartments were separated by removable dividers (Fig. 1D). The apparatus was filled with 3 L water and five zebrafish (three females, two males) were placed in one of the end-compartments, while the other end-compartment remained empty. The target fish used in this test originated from the F1 generation, were size-matched to the tested individuals and were not infected with *P. neurophilia*. A total of 18 *P. neurophilia*-infected and 18 uninfected fish was tested. Fish were individually placed in the central compartment and allowed to acclimate for 5 mins, after which the transparent dividers were removed. The fish’s behaviour was then video-recorded for 6 mins. In between tests, water was changed and the right/left location for the target fish was alternated in order to avoid lateral bias. Time spent in each compartment and number of crossings between compartments were quantified in order to establish a proxy for social preference in infected and non-infected zebrafish (following methodology by Miller & Gerlai^67^).

### Video analysis

Videos were manually analysed by a researcher blinded to the knowledge of specific treatments in order to avoid any bias. Biting (mirror biting test), number of entries to a zone (social preference test, light/dark preference test) and time spent freezing (open field test) were quantified manually. All zebrafish behaviour was furthermore tracked and quantified using Ethovision XT 13 (Noldus, Wageningen, The Netherlands).

### Statistics

We conducted all statistical analysis in the R Statistical Environment v3.2.4^68^, using the packages “lme4”, “MASS”, “pscl”, “multcomp”, “MuMin”, and “car”. For all models, to check that assumptions concerning normality and homoscedasticity were met, residual and quantile-quantile plots were inspected visually. For data that did not meet these assumptions, alternative distributions were used, as outlined below. Each model´s complete statistical output and R^2^ are included in the supplementary material (Table S1).

All measurements of size and condition (weight, length, Fulton´s K condition factor) as well as total distance moved (social preference test) were analysed using generalized linear models, with treatment (infected, uninfected), sex (male, female) and their interaction included as explanatory variables. For the proportion of time spent with conspecifics (social preference test), number of bites at the mirror image (mirror bite test), and time in the centre (open field test), a negative binomial generalized linear model was used (to address overdispersion and non-normal distribution in the data), with treatment, sex and their interaction included as explanatory variables. Freezing behaviour (in the mirror bite and open field tests) was analysed using a negative binomial generalized linear mixed-effects model (to address overdispersion and non-normal distribution in the data), with treatment, sex, test (mirror bite, open field) and all associated interactions included as fixed effects and individuals included as a random effect (due to repeated measures). Crossings between compartments and time spent in the dark (light/dark preference test) were analysed using zero-inflated count data regression models (to address the high proportion of zeros for these traits), with treatment, sex and their interaction included as explanatory variables. Significant interaction effects were followed by Tukey’s multiple comparisons post-hoc tests in order to ascertain significant differences between all groups.

## Supplementary information

Supplementary Information.

## Data Availability

The datasets generated during and analysed during the current study are available in the NMBU Open Reseach Data repository, [https://dataverse.no/dataset.xhtml?persistentId=doi:10.18710/OD7M8N].
