# Supplementary material for: Behavioural effects of the common brain-infecting parasite Pseudoloma neurophilia in laboratory zebrafish (Danio rerio)
Source: Sci Rep. 2020 May 15;10:8083. doi: 10.1038/s41598-020-64948-8 (PMC7228949; doi:10.1038/s41598-020-64948-8)
Supplement: Supplementary file 1 — Supplementary Information. [file 41598_2020_64948_MOESM1_ESM.docx]

Behavioural effects of the common brain-infecting parasite *Pseudoloma neurophilia* in laboratory zebrafish (*Danio rerio*)

Helene L.E. Midttun*, Marco A. Vindas, Lauren E. Nadler, Øyvind Øverli and Ida B. Johansen

**Supplementary data**

**
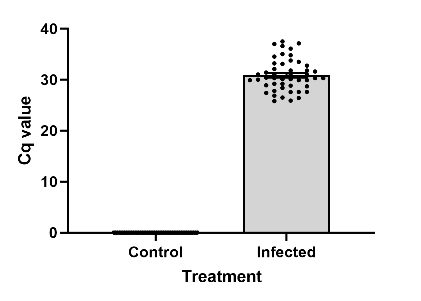
**

**Fig. S1.** Cq-values from qPCR-testing of zebrafish (*Danio rerio*) brains for the presence of the microsporidian parasite *Pseudoloma neurophilia*. Approximately 80% of all fish used for behavioural tests were screened for *P. neurophilia* and samples with Cq-values between 15-38 were considered positive for *P. neurophilia*. All fish in the infection treatment showed Cq-values between 15-38, none of the control fish showed any observable Cq-values. n_control_ = 51, n_infected_ = 47.

**Table S1**. Effect tests assessing variation in body size/condition and behaviour with infection by the microsporidium parasite *Pseudoloma neurophilia* in the zebrafish (*Danio rerio*). Generalized linear model analysis (gaussian distribution) was used to assess the role of infection treatment (uninfected, infected) and sex (male, female), and their interaction in individual size/condition and total distance moved (social preference test)

| **Test** | **Trait** | **Sample size** | **Factor** | **d.f.** | **F-value** | **p-value** | **R^2^** |
| --- | --- | --- | --- | --- | --- | --- | --- |
| Size & Condition | Weight (g) | Control= 57  Infected= 60 | Treatment | 1, 115 | 14.41 | 0.0002 | 0.47 |
|  |  |  | Sex | 1, 114 | 87.47 | < 0.0001 |  |
|  |  |  | Treatment*Sex | 1, 113 | 00.35 | 0.319 |  |
|  | Length (cm) | Control= 57  Infected= 60 | Treatment | 1, 115 | 20.55 | < 0.0001 | 0.23 |
|  |  |  | Sex | 1, 114 | 13.73 | 0.0003 |  |
|  |  |  | Treatment*Sex | 1, 113 | 3.92 | 0.597 |  |
|  | Fulton’s K condition factor | Control= 57  Infected= 60 | Treatment | 1, 115 | 6.42 | 0.467 | 0.36 |
|  |  |  | Sex | 1, 114 | 62.91 | < 0.0001 |  |
|  |  |  | Treatment*Sex | 1, 113 | 5.08 | 0.178 |  |
| Social Preference | Total distance moved (cm) | Control= 18  Infected= 18 | Treatment | 1, 34 | 5.490 | 0.026 | 0.20 |
|  |  |  | Sex | 1, 33 | 1.746 | 0.196 |  |
|  |  |  | Treatment*Sex | 1, 32 | 1.392 | 0.347 |  |

**Table S2.** Effect tests assessing behaviour with infection by the microsporidium parasite *Pseudoloma neurophilia* in the zebrafish (*Danio rerio*). The role of infection treatment and sex (and their interaction) in zebrafish behaviour. The proportion of time spent with conspecifics, number of bites at the mirror image and time spent in the centre were analysed using a generalized linear model with a negative binomial distribution. Freezing was assessed using a generalized linear mixed-effects model with test (mirror bite, open field) as an additional fixed effect and individual as a random effect. All factors from the light/dark preference test (crossings between compartments, time spent in dark) were analysed using a zero-inflated count data regression model. The sample size and R^2^ for each model are indicated.

| **Test** | **Trait** | **Sample size** | **Factor** | **d.f.** | **χ^2^** | **p-value** | **R^2^** |
| --- | --- | --- | --- | --- | --- | --- | --- |
| Social Preference | Proportion of time spent with conspecifics | Control= 18  Infected= 18 | Treatment | 1 | 0.17 | 0.681 | 0.12 |
|  |  |  | Sex | 1 | 0.02 | 0.899 |  |
|  |  |  | Treatment*Sex | 1 | 2.18 | 0.139 |  |
| Mirror Bite | Number of bites at mirror image | Control= 18  Infected= 17 | Treatment | 1 | 0.49 | 0.485 | 0.05 |
|  |  |  | Sex | 1 | 0.30 | 0.586 |  |
|  |  |  | Treatment*Sex | 1 | 0.00 | 0.991 |  |
| Open Field | Time spent in centre (s) | Control= 18  Infected= 18 | Treatment | 1 | 2.77 | 0.096 | 0.10 |
|  |  |  | Sex | 1 | 0.23 | 0.634 |  |
|  |  |  | Treatment*Sex | 1 | 3.92 | 0.047 |  |
| Mirror Bite/Open Field | Freezing | Control= 18  Infected= 17 | Test | 1 | 2.10 | 0.148 | 0.61 (R^2^m), 0.99 (R^2^c) |
|  |  |  | Treatment | 1 | 5.40 | 0.020 |  |
|  |  |  | Sex | 1 | 3.68 | 0.102 |  |
|  |  |  | Test*Treatment | 1 | 1.56 | 0.212 |  |
|  |  |  | Test*Sex | 1 | 0.29 | 0.590 |  |
|  |  |  | Treatment*Sex | 1 | 0.28 | 0.600 |  |
| Light/Dark Preference | Crossings between compartments | Control= 18  Infected= 18 | Treatment | 1 | 9.73 | 0.002 | 0.23 (Nagelkerke pseudo-R^2^) |
|  |  |  | Sex | 2 | 0.99 | 0.610 |  |
|  |  |  | Treatment*Sex | 2 | 1.29 | 0.525 |  |
|  | Time spent in dark | Control= 18  Infected= 18 | Treatment | 1 | 0.72 | 0.396 | 0.10  (Nagelkerke pseudo-R^2^) |
|  |  |  | Sex | 2 | 0.62 | 0.734 |  |
|  |  |  | Treatment*Sex | 2 | 0.01 | 0.997 |  |


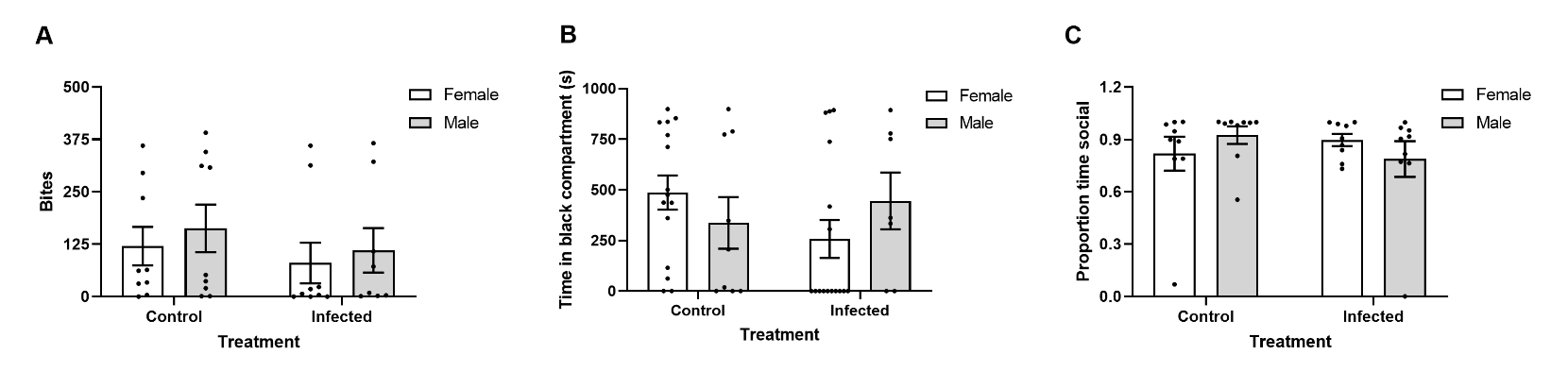


**Fig. S2**. Non-significant behavioural effects of *Pseudoloma neurophilia*-infection in female and male experimentally infected zebrafish and uninfected controls. **A** Total amount of bites at own mirror image in the mirror biting test (bites ± SEM), p = 0.485, n_control_ = 18, n_infected_ = 17. **B** Total time spent in the black compartment in the Light/Dark preference test (s ± SEM), p = 0.396, n = 25 per treatment group. **C** Proportion of time in close proximity to conspecifics in the social preference test (proportion ± SEM), p = 0.681, n = 18 per treatment group. All statistical analysis performed in the R Statistical Environment v3.2.4, ^44^, graphs obtained using GraphPad Prism v8.3.1^45^.
